# Capgras Syndrome as a Manifestation of a Neurodegenerative Disease – What do we know?

**DOI:** 10.1192/j.eurpsy.2023.780

**Published:** 2023-07-19

**Authors:** M. Matias, L. Lopes, I. Grenha, M. Marques, M. Alves

**Affiliations:** Unidade Local de Saude Alto Minho, Viana do Castelo

## Abstract

**Introduction:**

The Capgras syndrome (CS), firstly described in 1899, is a delusional conviction that a person emotionally close has been replaced by an imposter or duplicate. It has been associated to primary psychiatric disturbances as well as neuropsychiatric syndromes. Its etiology and management have been debated throughout the years. We describe a case of a 75 years old male who was admitted to our psychiatric ward due to aggressiveness towards his spouse, believing she was an imposter.

**Objectives:**

In light of this case, we aim to discuss its etiology and review the association between the Capgras syndrome and neurodegenerative diseases.

**Methods:**

Classically, CS was associated to psychotic illnesses such as schizophrenia, schizoaffective disorder and substance abuse. However, recent studies shed light on other possible etiologies, such as neurodegenerative and nonneurodegenerative diseases. In older ages, it has been associated to Alzheimer’s and, most commonly, Lewy body dementia subtype. Research also shows that other misidentification syndromes are frequently present in association with CS. Patients are more likely to be aggressive towards caregivers under these circumstances. Studies suggest there is a higher prevalence of right hemisphere lesions in CS, namely frontal and temporal lobes, that impair facial processing. Various brain circuits are being proposed as possible etiopathogenesis.

In this case, parkinsonian signs were observed in our patient, such as resting tremor, imbalance gait and rigidity. Those had not been described before his hospitalization. His family stated memory loss and difficulty in executive functions were present for at least a year. This patient had no previous psychiatric history. Brain CT scan showed cortical atrophy.

**Results:**

A neurodegenerative cause was assumed, and the patient was started on a cholinesterase inhibitor and on a second-generation antipsychotic. Improvement was observed.

**Conclusions:**

This case is an example of the heterogenous etiology of the CS. It is important to consider different diagnosis, especially in older ages. More studies are needed to improve the knowledge on CS etiopathogenesis as well as the brains circuits involved. Psychopharmacology tackling theses syndromes is also a growing.

**Disclosure of Interest:**

None Declared

